# The *MdAux/IAA2* Transcription Repressor Regulates Cell and Fruit Size in Apple Fruit

**DOI:** 10.3390/ijms23169454

**Published:** 2022-08-21

**Authors:** Haidong Bu, Xiaohuan Sun, Pengtao Yue, Junling Qiao, Jiamao Sun, Aide Wang, Hui Yuan, Wenquan Yu

**Affiliations:** 1 Mudanjiang Branch, Heilongjiang Academy of Agricultural Sciences, Mudanjiang 157000, China; 2 Key Laboratory of Fruit Postharvest Biology (Liaoning Province), Key Laboratory of Protected Horticulture (Ministry of Education), National & Local Joint Engineering Research Center of Northern Horticultural Facilities Design & Application Technology (Liaoning), College of Horticulture, Shenyang Agricultural University, Shenyang 110866, China

**Keywords:** apple, fruit size, auxin, *MdAux/IAA2*, cell size

## Abstract

Auxin plays an important role in regulating plant development, and *Auxin/indole acetic acid* (*Aux/IAA*) is a type of auxin-responsive gene and plays an important role in auxin signaling; to date, although 29 Aux/IAA proteins have been reported in *Abrabidopsis thaliana*, only parts of the *Aux/IAA* family gene functions have been identified. We previously reported that a bud sport of ‘Longfeng’ (LF) apple (*Malus domestica*), named ‘Grand longfeng’ (GLF), which showed a larger fruit size than LF, has lower expression of *MdAux/IAA2*. In this study, we identified the function of the *MdAux/IAA2* gene in apple fruit size difference using *Agrobacterium*-mediated genetic transformation. Overexpression of *MdAux/IAA2* decreased the apple flesh callus increment and caused a smaller globular cell size. In addition, overexpression of *MdAux/IAA2* in GLF fruit resulted in the reduction of apple fruit size, weight, and cell size, while silencing *MdAux/IAA2* in LF apple fruit resulted in an increase in apple fruit weight and cell size. We suggest that the high auxin content depressed the expression of *MdAux/IAA2*, and that the downregulated expression of *MdAux/IAA2* led to the formation of GLF. Our study suggests a mechanism for fruit size regulation in plants and we will explore the transcription factors functioning in this process in the future.

## 1. Introduction

Fruit size (quantitative traits controlled by multiple genes) is a major agronomic trait that influences the quality and economic value of apple (*Malus domestica*) [1,2]. Phytohormones affect plant development, including embryo occurrence and lateral branch formation [3,4]. For example, the cell number can be increased by cytokinin such as BA (benzyladenine), which increased the fruit size in the ‘Empire’ apple by inducing a greater number of cells in the fruit cortex [5]. Gibberellin treatment increased the ‘Kosui’ pear fruit size [6]. Gibberellin increased the fruit size and enhanced the fruit elongation and fruit shape index in apple [7]. Auxin is another important phytohormone that affects fruit size. Auxin increased the fruit size by increasing the cell size, but it did not change the fruit shape in the ‘Royal Gala’ apple [8]. Auxin synthesis, transport, metabolism, and signal transduction are synergistic and complex processes in plants [9,10]. Auxin synthesis is mainly determined by two main enzymes, tryptophan aminotransferase of Arabidopsis/tryptophan aminotransferase related (TAA1/TAR) and flavin monooxygenase (YUCCA) in plants [11]. Then, auxin is transported by auxin resistant 1/like auxin resistant 1 (AUX1/LAX1) and PIN-formed 1 (PIN1) from extracellular to intracellular and in the reverse direction after auxin synthesis, respectively [12]. In addition to its synthesis and transport, auxin is degraded by gretchen hagen 3 (GH3) family protein [8]. Changes in the endogenous auxin content led to changes in the expression of auxin-responsive genes [13]; however, the underlying mechanisms of how auxin regulates fruit size are poorly understood.

The auxin early response family gene contains *Auxin/indole acetic acid* (*Aux/IAA*), *GH3* (*Gretchen Hagen3*), and *SAUR* (*Small auxin up* RNA), and *Aux/IAA* is one of the three major member genes [8]. Twenty-nine Aux/IAA proteins have been identified in *Arabidopsis thaliana* [14]. Aux/IAA can interact with the auxin receptor transport inhibitor response 1/auxin signaling F-BOX protein (TIR1/AFB, a component of E3 ubiquitin ligase). When the auxin content is high, Aux/IAA is degraded by TIR1/AFB and releases auxin response factors (ARFs), which are components of Aux/IAA and ARF heterodimers, leading to the activation of auxin signaling [15,16]. Conversely, ARFs are locked by Aux/IAA, and auxin signaling is depressed [17].

*Aux/IAAs* are involved in diverse plant growth and development processes; for example, the silencing of *SlAux/IAA15* decreases apical dominance and trichome number, resulting in more green leaves and lateral roots [18]. The silencing of *SlAux/IAA9* initiates the fruit setting process and regulates leaf morphogenesis in tomato [19]. In addition, Aux/IAA functions in regulating fruit size in tomato and decreasing the expression of *SlAux/IAA27* resulted in a smaller fruit size and lower fertility compared with the control [20]. However, the silencing of the *SlAux/IAA17* gene resulted in an increase in cell size and produced a significantly larger fruit size [21]. These studies indicate specialized roles for *Aux/IAAs* in plant developmental processes, clearly indicating that members of the *Aux/IAA* gene family in fruit performs both overlapping and specific functions.

The expression levels of *FaAux/IAA1* and *FaAux/IAA2* are negatively correlated with auxin levels in strawberry [22]. However, the detailed function of Aux/IAA has not been verified in fruit trees, and the relationship between auxin and fruit size is poorly understood. Our previous results suggest that increases in auxin levels were the reason for the bud sport variety ‘Grand Longfeng’ (GLF) fruit size being significantly larger than that of LF apple. Moreover, two Aux/IAAs (*MdAux/IAA2* and *MdAux/IAA26*) were downregulated in the GLF fruit compared to LF [23].

In this research, we analyzed the transcription levels of *MdAux/IAA2* and *MdAux/IAA26* in fruit development stages and revealed that *MdAux/IAA2* expression showed a reverse trend with fruit and cell size. Moreover, 1-naphthylacetic acid (NAA) treatment significantly decreased the expression of *MdAux/IAA2*, and 2,3,5-triiodobenzoic acid (TIBA) treatment significantly increased the expression of *MdAux/IAA2*. The function of *MdAux/IAA2* in regulating fruit size was studied.

## 2. Results

### 2.1. Expression of MdAux/IAA2 in GLF Was Lower Than That in LF Fruit

**Figure 1:**
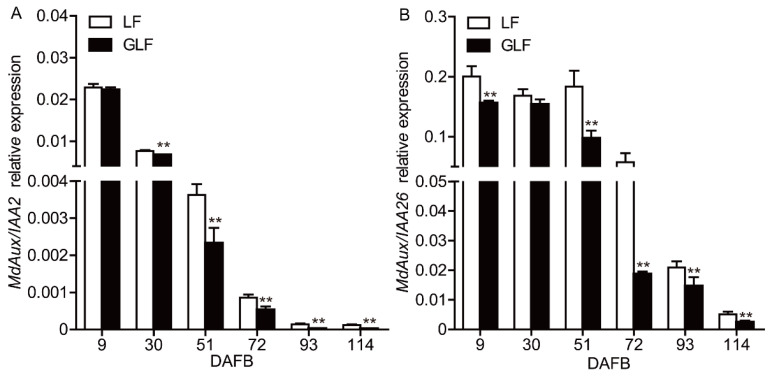
Expression of *MdAux/IAA2* and *MdAux/IAA26* in LF and GLF fruit. qRT-PCR was used to measure the relative expression of *MdAux/IAA2* (**A**) and *MdAux/IAA26* (**B**) in the GLF and LF fruit cortexes from 9 to 114 days after full bloom (DAFB). *MdActin* gene (housekeeping; EB136338) was used as endogenous control. ** Significant differences (*p* < 0.05, Student’s *t*-test). Error bars indicate the standard deviation (SD) of the three biological replicates. LF: Longfeng; GLF: Grand Longfeng.

Previous results revealed that the fruit size of GLF, a bud sport variety of ‘Longfeng’, was larger than that of LF, and two auxin-responsive genes (*MdAux/IAA2* and *MdAux/IAA26*) showed downregulated expression in GLF compared with LF at 51, 72, and 93 DAFB [23]. In this study, the expressions of *MdAux/IAA2* and *MdAux/IAA26* were measured throughout the fruit developmental period (9, 30, 51, 72, 93, and 114 DAFB). The expression of *MdAux/IAA2* in GLF was significantly lower than that in LF from 30 to 114 DAFB, showing no significant difference at 9 DAFB (Figure 1A), which coincided with the periods when the auxin level, fruit phenotype, and cell size began to show differences between the two varieties [23]. However, the expression of *MdAux/IAA26* was lower in GLF than in LF at 9, 51, 72, 93, and 114 DAFB, and there was no significant difference at 30 DAFB (Figure 1B). This time span showed a lack of conformity with the periods when the auxin level, fruit phenotype, and cell size began to show differences between the two varieties (Figure 1B) [23].

### 2.2. Exogenous Auxin Inhibited the Expression of MdAux/IAA2

**Figure 2:**
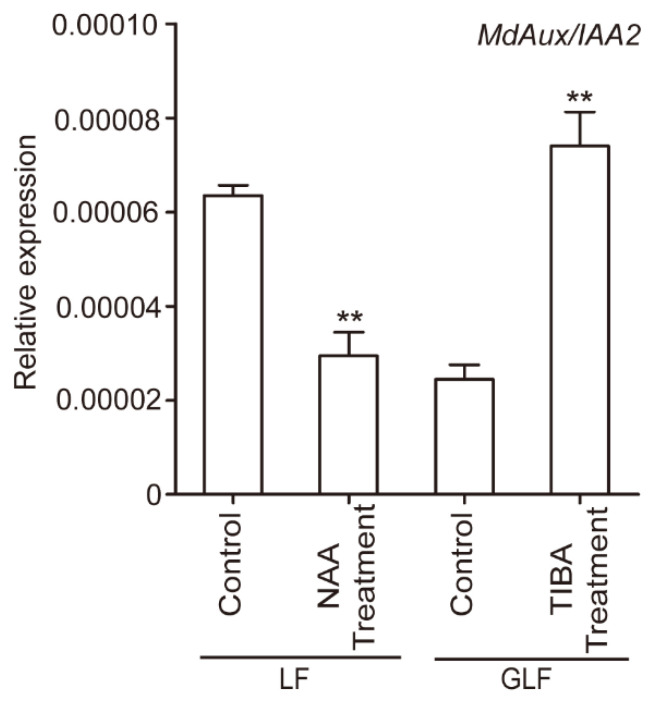
The influence of auxin and TIBA on the *MdAux/IAA2* gene in fruit. *MdActin* gene (EB136338) was used as endogenous control. ** Significant differences (*p* < 0.05, Student’s *t*-test). Error bars indicate the standard deviation (SD) of the three biological replicates. LF: Longfeng; GLF: Grand Longfeng.

To determine whether the auxin content affected the transcription level of *MdAux/IAA2*, qRT-PCR was previously used to analyze the transcription level of *MdAux/IAA2* in apple fruit treated with 1-naphthylacetic acid (NAA) and 2,3,5-triiodobenzoic acid (TIBA, an inhibitor of auxin transport polarity) [23]. Interestingly, NAA treatment significantly decreased the transcription level of *MdAux/IAA2* in LF (Figure 2). Moreover, TIBA treatment significantly increased the transcription level of *MdAux/IAA2* in GLF (Figure 2). Combined with the previous results, that NAA treatment significantly increased the LF apple fruit and cell size, TIBA treatment significantly decreased the GLF apple fruit and cell size [23]. These results suggest that *MdAux/IAA2* might play an important role in regulating apple fruit size.

### 2.3. MdAux/IAA2 Is a Nuclear Location Protein

To determine whether there was a difference in the *MdAux/IAA2* sequence between LF and GLF, the coding sequences (CDS) and promoter of the 1550-bp longer *MdAux/IAA2* were cloned from cDNA and gDNA of LF and GLF, respectively. The results revealed that the CDS sequence of *MdAux/IAA2* was 888 bp. Moreover, the CDS and promoter of *MdAux/IAA2* in GLF were the same those as in LF (Figures S1 and S2).

**Figure 3:**
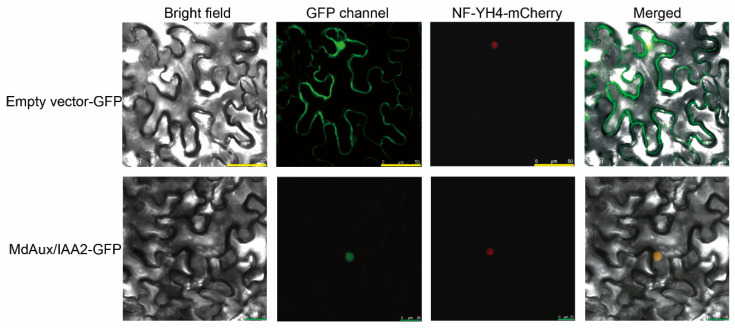
Subcellular localization of MdAux/IAA2. NF-YA4-mCherry was the nuclear marker. MdAux/IAA2-GFP was MdAux/IAA2, which was connected to the carrier of PRI101 containing the GFP label. The empty vector-GFP was the control. The empty vector-GFP yellow scale was 50 μm, and the MdAux/IAA2-GFP green scale was 25 μm.

Aux/IAA family proteins contain conserved nuclear localization signals [24,25]. To determine the subcellular localization of MdAux/IAA2, the CDS region of *MdAux/IAA2* was connected to the pRI101 vector containing GFP to construct MdAux/IAA2-GFP, then, MdAux/IAA2-GFP was injected in tobacco leaves (*N. benthamiana*) using *Agrobacte*-rium-mediated transformation, and the subcellular localization of MdAux/IAA2 was analyzed with empty vector GFP as the control. The results revealed that MdAux/IAA2 was a nuclear localization protein (Figure 3).

**Figure 4:**
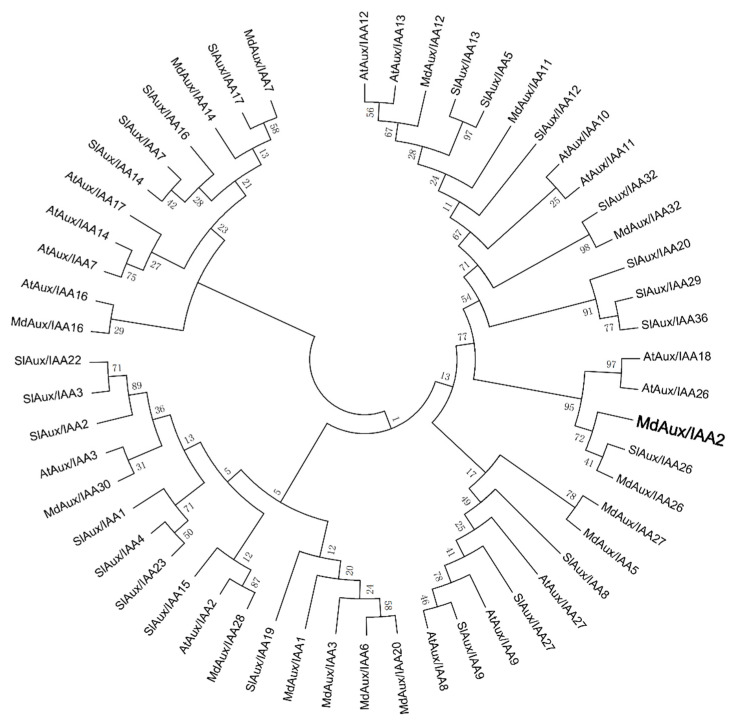
Phylogenetic tree analysis of MdAux/IAA2.The amino acid sequences of the Aux/IAA family in *Arabidopsis thaliana*, tomato, and apple were downloaded, and phylogenetic trees were constructed using the neighbor-joining method of MEGA7 software. Bold part is MdAux/IAA2.

Some of the Aux/IAA family gene functions have been reported. Through analysis of the relationship of the MdAux/IAA2 protein with other Aux/IAAs, phylogenetic tree analysis revealed that MdAux/IAA2 had a closer genetic relationship with SlAux/IAA26 of tomato and MdAux/IAA26 of apple (Figure 4).

### 2.4. Overexpression of MdAux/IAA2 Decreases the Fruit Flesh Callus

To clarify the function of *MdAux/IAA2*, we overexpressed it in the apple flesh callus by *Agrobacterium*-mediated genetic transformation (MdAux/IAA2-OE) (Figure S3). The transcription level of *MdAux/IAA2* in overexpression lines (MdAux/IAA2-OE1, MdAux/IAA2-OE2, MdAux/IAA2-OE3) was significantly higher than that of the control (empty vector) after 20 days of culture (Figure S3A). Moreover, the increments of MdAux/IAA2-OE1, MdAux/IAA2-OE2, and MdAux/IAA2-OE3 were lower than those of the empty vector (Figure S3B), and the global cell size in MdAux/IAA2-OE was smaller than that of the control.

### 2.5. MdAux/IAA2 Negatively Regulated Apple Fruit Size

**Figure 5:**
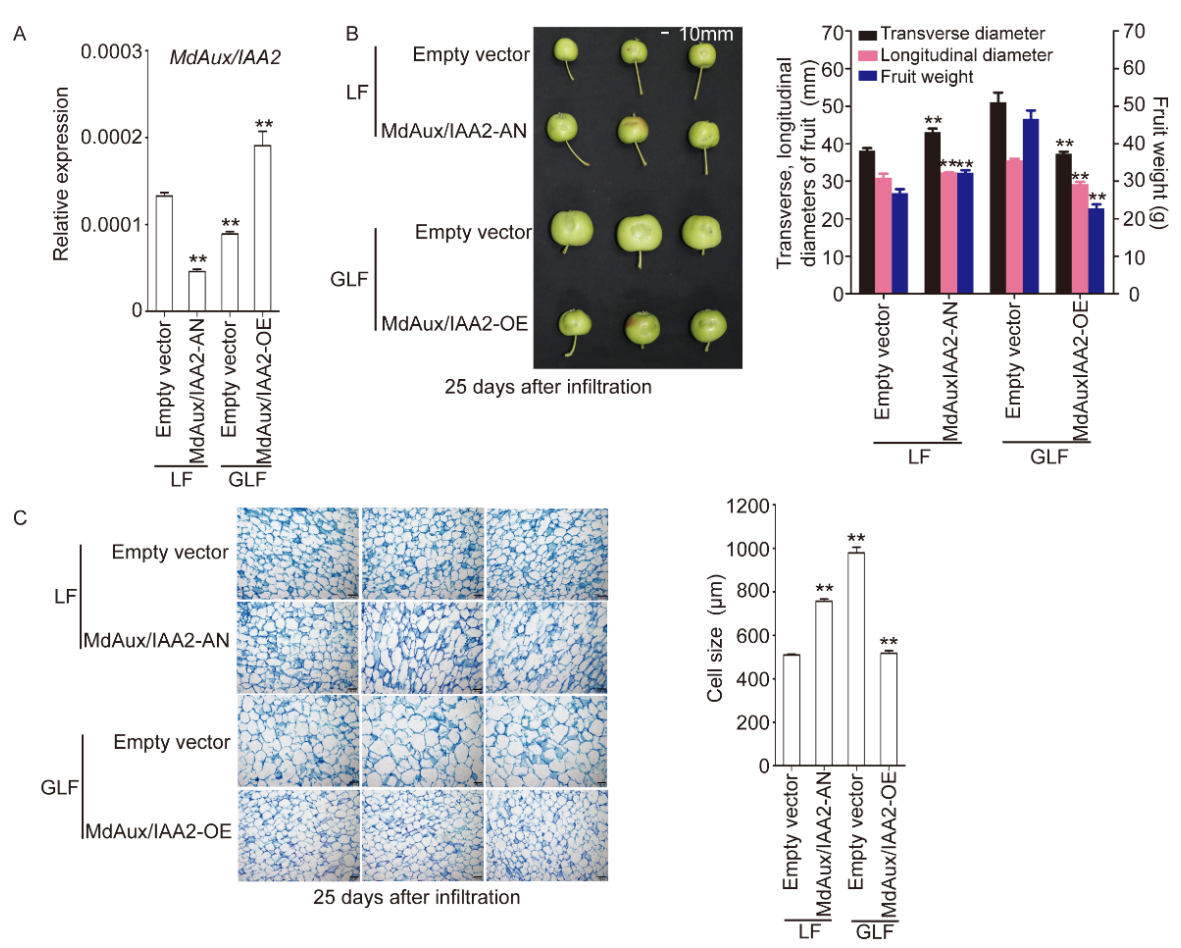
Functional identification of the MdAux/IAA2 gene. The expression of *MdAux/IAA2* was measured in MdAux/IAA2-AN and MdAux/IAA2-OE fruit using qRT-PCR, *MdActin* gene (EB136338) was used as endogenous control; error bars indicate the standard deviation (SD) of three biological replicates (**A**). Fruit transversal diameter, longitudinal diameter, and weight were measured using a digital Vernier caliper and electronic scales 25 days after fruit injection; bar = 10 mm, error bars indicate the standard deviation (SD) of 10 biological replicates (**B**). Fruit cell and quantitative results in MdAux/IAA2-AN and MdAux/IAA2-OE apple fruit; error bars indicate the standard deviation (SD) of three biological replicates (**C**). Cell size was the average of six typical cell lengths, which were measured by a scale tool in the microscope 25 days after fruit injection; bar = 100 μm. Empty vector, pRI101-overexpressing fruit. ** Significant differences (*p* < 0.05, Student’s *t*-test).

To better understand the function of *MdAux/IAA2* in apple fruit, we overexpressed *MdAux/IAA2* (MdAux/IAA2-OE) in the GLF apple fruit (Figure 5). The transcriptional level of *MdAux/IAA2* was investigated 25 days after infiltration, and it was strongly increased in MdAux/IAA2-OE fruit compared with the control (Figure 5A). MdAux/IAA2-OE fruit displayed a smaller size compared with the control, and the transversal and longitudinal diameters and fruit weight decreased by 1.37-, 1.22-, and 2.06-fold, respectively, compared with the control (Figure 5B). In addition, the cell size of MdAux/IAA2-OE GLF fruit was smaller than that of the control (Figure 5C). Based on the above results, we suggest that *MdAux/IAA2* negatively regulates apple fruit size.

We then silenced the *MdAux/IAA2* gene (MdAux/IAA2-AN) in LF apples at 30 DAFB using an *Agrobacterium*-mediated transient genetic transformation (Figure 5). MdAux/IAA2-AN fruit showed significant downregulation of *MdAux/IAA2* compared with the control (Figure 5A). MdAux/IAA2-AN fruit displayed a larger fruit size, and the transversal and longitudinal diameters and fruit weight increased 1.13-, 1.05-, and 1.20-times, respectively, compared with the control (Figure 5B). Moreover, the cell size of the MdAux/IAA2-AN fruit flesh in GLF was much larger than that in the control (Figure 5C). Overall, these results suggest that *MdAux/IAA2* negatively regulates apple fruit and cell size.

## 3. Discussion

Fruit size is a major trait that influences the quality and economic value of apple [1,2]. Ploidy, genetic control, and hormone levels can influence fruit size [8,26,27]. Auxin synthesis, binding, and transport synergistically regulate auxin levels in plants [28]. A change in the auxin content can cause an auxin signal response in vivo [13].

*Aux/IAA* is a major auxin response gene family that includes *Gretchen Hagen3* (*GH3*) and *Small auxin up RNA* (*SAUR*) [8]. The transcriptional levels of *FaAux/IAA1* and *FaAux/IAA2* are negatively regulated by auxin in strawberry [22]; however, the gene function has not been reported in fruit trees. In this study, the transcriptional level of *MdAux/IAA2* in GLF was significantly lower than that in LF (Figure 1A). In addition, exogenous auxin inhibited the expression of *MdAux/IAA2*, but auxin inhibitor TIBA promoted the expression of *MdAux/IAA2* (Figure 2), which suggests that *MdAux/IAA2* may be negatively correlated with the auxin content. We overexpressed the *MdAux/IAA2* gene in the fleshy callus, and the increase in *MdAux/IAA2* overexpression was significantly less than that in the control (Figure S3). Moreover, the overexpression of *MdAux/IAA2* in the GLF fruit significantly decreased fruit and cell size and silencing of *MdAux/IAA2* in the LF fruit significantly increased the fruit and cell size (Figure 5). Overall, *MdAux/IAA2* negatively regulated apple fruit and cell size.

In tomato, silencing of the *SlAux/IAA17* gene increased fruit size [21]. However, the gene function of *SlAux/IAA17* was the same as *MdAux/IAA2* but silencing of the *SlAux/IAA27* gene decreased the fruit size and was different from that of *MdAux/IAA2* [20], indicating that Aux/IAA family genes have differential functions in flesh fruit regulation. In addition, phylogenetic tree analysis revealed that MdAux/IAA2 was not in the same branch as SlAux/IAA17 [21] and SlAux/IAA27 [20] but was in the same branch as SlAux/IAA26 of tomato, MdAux/IAA26 of apple, and AtAux/IAA26 and AtAux/IAA18 of *Arabidopsis thaliana* (Figure 3). However, these gene function have not been reported. Therefore, the function of the *MdAux/IAA2* gene in influencing apple fruit size has been found for the first time.

Apple fruit size is a quantitative trait that is influenced by multiple genes [29,30], and the expression of *Aux/IAA* can be regulated by transcriptional factors. bZIP11 inhibits bud and root growth by promoting *Aux/IAA3/SHY2* (a key negative regulator of root growth) transcription in *Arabidopsis thaliana* [31]. To investigate whether *MdAux/IAA2* could be regulated by transcription factors, we analyzed the promoter of *MdAux/IAA2* and found that trans-acting binding sites of transcription factors HY5, MYB, ARF, bZIP, and bHLH exist in the promoter of *MdAux/IAA2* (Figure S4). Combined with transcriptome analysis [23], in addition to MdARF5, there were five upregulated and 10 downregulated MYB genes (Table 1), six upregulated bZIP genes (Table 2), and 11 downregulated bHLH genes (Table 3). How transcription factors regulate auxin synthesis and signaling and, thus, regulate cell and fruit size during the GLF and LF fruit development processes will become the focus of further research.

**Figure 6:**
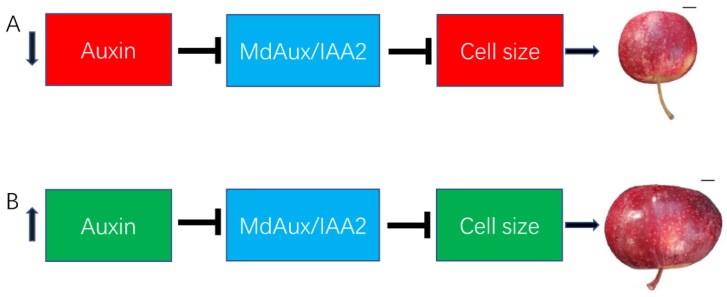
A working model of *MdAux/IAA2* for regulating apple fruit size. A model role of MdAux/IAA2 in auxin-mediated differences in fruit size between LF and GLF. *MdAux/IAA2* negatively regulates apple fruit and cell size; thus, in the absence of auxin, MdAux/IAA2 is enhanced, and the LF apple has a small cell size and fruit size. Bar, 10 mm (**A**). When the auxin content is high, MdAux/IAA2 may be degraded by TIR1/AFB (a component of E3 ubiquitin ligase), and the negative regulation of MdAux/IAA2 in apple cell size was eliminated, resulting in the larger fruit size of GLF. Bar, 10 mm (**B**).

A model of how *MdAux/IAA2* regulates apple cell size and fruit size is shown in Figure 6. When the auxin level was low, *MdAux/IAA2* expression increased, and most MdAux/IAA2 accumulated in the apple fruit. *MdAux/IAA2* negatively regulated the cell size, which led to a smaller fruit size in LF (Figure 6A). When the auxin content was high, TIR1/AFB degraded MdAux/IAA2 by ubiquitination via E3 ubiquitin ligase, and the negative regulation effect of MdAux/IAA2 on apple fruit size decreased, leading to a larger cell size and fruit size in apple (GLF) (Figure 6B).

## 4. Materials and Methods

### 4.1. Plant Materials and Treatment

Longfeng and ‘Grand Longfeng’ apple (*Malus domestica*) trees were grown on *M. baccara* rootstocks at an orchard (E 129°32′12″, N 44°18′00″) in Dongsheng Village, Ningan Town, Mudanjiang city, Heilongjiang Province, China. Phenotypic and cytological identification of the fruit was carried out as previously described [23]. Three transgenic and empty vector callus or ten typical pieces of fruit were frozen in liquid nitrogen and stored at −70 °C for RNA extraction.

### 4.2. Gene Cloning and Bioinformatics Analysis

RNA extraction and cDNA synthesis were the same as previously described [23]. PCR (Polymerase Chain Reaction) was performed on a PCR system (Analytik Jena, Jena, Germany) with a 10 μL total volume containing 5 mL SYBR Green Master Mix (Cat. 04707516001; Roche Diagnostic Ltd., Basel, Switzerland), 0.5 mL cDNA, 0.5 mL reverse and forward primers, and 3.5 mL H_2_O. The reaction program was as follows: 98 °C for 5 min, followed by 35 cycles of 9 s at 98 °C, 15 s at 55 °C, 90 s at 72 °C, and a final 5 min at 72 °C. Then, 3 μL dNTP mixture and 0.5 μL R Tap were added to the reactions at 72 °C for 20 min. The full-length *MdAux/IAA2* gene was detected by 1% agarose gel electrophoresis. Plasmid recovery and seamless cloning were performed following Yue et al. [32].

### 4.3. Vector Construction of MdAux/IAA2

The seamless cloning kit (Cat. No. D7010M, Beyotime, Shanghai, China) was used, following Yue et al. [32]. The CDS region of *MdAux/IAA2* was connected to the pRI101 vector containing green fluorescent protein (GFP) to construct MdAux/IAA2-GFP. MdAux/IAA2-GFP was injected in tobacco leaves (*N. benthamiana*) using *Agrobacterium*-mediated transformation, and empty vector-GFP as control, and NF-YA4-mCherry was the nuclear marker. MdAux/IAA2-OE (restriction sites were *Nde*I and *Sal*I), and MdAux/IAA2-AN (restriction sites were *Sal*I and *Nde*I) with the pRI101 vector were constructed for overexpression and silencing of the MdAux/IAA2 protein using the seamless cloning kit (Cat. No. D7010M, Beyotime, Shanghai, China). An empty vector was used as the control. All primers are listed in Table 4.

### 4.4. Callus and Fruit Transformation

The fruit flesh callus was obtained from ‘Orin’ apple fruit and carried out following Alayón-Luaces et al. [33]. Fruit flesh callus was cultured on Murashige and Skoog (MS) medium (M519-100, Phytotech, Lenexa, Kansas, United States) with 1.5 mg L^−1^ 6-butyric acid (BA; Sigma-Aldrich, Poole, UK) and 0.5 mg L^−1^ indole acetic acid (Sigma-Aldrich, Poole, UK) at 25 °C in the dark. The overexpression vector (MdAux/IAA2-OE) of MdAux/IAA2 was transformed into 20-day-old ‘Orin’ callus tissue using *Agrobacterium tumefaciens* strain EHA105, according to An et al. [34]. The empty vector was used as a control. Samples of MdAux/IAA2-OE and empty vector callus were sub-cultured twice for 20 days each. The callus was stained with 1% toluidine blue for 5 min, and then the dye was removed. An Olympus BX50f-3 microscope (Olympus Optical Co., Ltd., Tokyo, Japan) was used to measure the globular cell size of the apple fruit callus. Each fruit callus was used as one biological replicate with a total of three biological replicates. Callus samples were frozen in liquid nitrogen and stored at −70 °C for RNA extraction.

For the *MdAux/IAA2* overexpression GLF apple, 1 mL of *Agrobacterium* suspension with the *MdAux/IAA2*-overexpressing vector (OD_600_ = 0.8) and empty vector (OD_600_ = 0.8) was injected into the GLF fruit at 30 days after full bloom (DAFB). For the *MdAux/IAA2*-silenced LF apple, 1 mL of *Agrobacterium* suspension with the *MdAux/IAA2*-silencing vector (OD_600_ = 0.8) and empty vector (OD_600_ = 0.8) was injected into the LF fruit at 30 DAFB. Fruit was injected with the empty vector as a control. Fruit was harvested 25 days after fruit injection. Each fruit was used as one biological replicate with a total of 10 biological replicates. Fruit samples were sliced, frozen in liquid nitrogen, and stored at −70 °C for RNA extraction.

Fruit flesh RNA extraction and cDNA synthesis were performed according to previously described methods. Quantitative reverse transcription-PCR (qRT-PCR) was performed according to previous methods [23]. 

### 4.5. Bioinformatics Analysis

Aux/IAA family FASTA protein sequences of *Arabidopsis thaliana*, tomato, and apple were obtained from the *Arabidopsis thaliana* (https://www.arabidopsis.org/; accessed on 1 June 2016), tomato (https://solgenomics.net/; accessed on 30 May 2012), and apple genome websites (https://iris.angers.inra.fr/gddh13/; accessed on 13 september 2016), and phylogenetic trees were constructed using MEGA7 software (Mega Limited, Auckland, New Zealand). The Plantcare online website (http://bioinformatics.psb.ugent.be/webtools/plantcare/html/; accessed on 1 January 2012) was used for the trans-acting factor prediction analysis.

### 4.6. Statisticical Analysis

Statistical analysis was performed using Student’s *t*-test in SPSS V18.0 (IBM, Chicago, IL, USA), and independent-samples T test of 95% as the confidence interval percentage was used for data analysis. Quantitative reverse transcription-PCR analysis in this study used three biological replicates in transgenic and empty vector callus for Student’s *t*-test analysis. LF, GLF, and 1-naphthylacetic acid (NAA) (NAA; BBI Life Sciences, Shanghai, China), 2,3,5-triiodobenzoic acid (TIBA, an inhibitor of auxin transport polarity; (TIBA; Shanghai Maokang Biotechnology Co., Ltd., Shanghai, China)) fruits were collected from three trees (three fruits per tree), and the flesh of each tree was equally mixed for qRT-PCR analysis, as previous described [23], for treatment of fruits. Fruit core diameter, longitudinal diameter, and transversal diameter were performed as in previous methods [23], ten fruits were used for statistical analysis of Student’s *t*-test. Cytological analysis was used as in previous methods [23].

## 5. Conclusions

In summary, we showed that the auxin signaling gene *MdAux/IAA2* is a negative regulator of fruit and cell size. High endogenous auxin content inhibited the expression of *MdAux/IAA2*, resulting in significantly greater fruit for GLF than for LF. This study provides a mechanism for fruit size regulation in plants.

## Figures and Tables

**Table 1:** Differentially expressed MYB transcription factors in LF and GLF fruit.

Gene ID	Gene Name	FDR (False Discovery Rate)	Log2FC	Relative Expression Mode
MD13G1083200	PREDICTED: transcription factor bHLH79-like [*Malus domestica*]	5.35948 × 10^−22^	−2.471969519	down
MD15G1377800	BHLH domain class transcription factor [*Malus domestica*]	1.05255 × 10^−11^	−1.505775683	down
MD02G1009200	PREDICTED: transcription factor bHLH66-like [*Malus domestica*]	8.88007 × 10^−12^	−1.096337386	down
MD10G1156100	PREDICTED: transcription factor bHLH62 [*Malus domestica*]	2.90976 × 10^−14^	−2.156869865	down
MD11G1173100	PREDICTED: transcription factor LHW-like [*Pyrus* × *bretschneideri*]	2.90818 × 10^−5^	−1.014574381	down
MD11G1230700	PREDICTED: transcription factor bHLH123-like isoform X1 [*Malus domestica*]	0.001677345	−1.383493476	down
MD07G1192000	PREDICTED: transcription factor bHLH111-like isoform X2 [*Malus domestica*]	3.31608 × 10^−6^	−1.349332523	down
MD12G1112400	PREDICTED: transcription factor bHLH36-like isoform X3 [*Malus domestica*]	0.001632799	−1.674088612	down
MD15G1023100	PREDICTED: transcription factor bHLH110-like isoform X1 [*Malus domestica*]	3.28979 × 10^−7^	−1.074724796	down
MD08G1026300	PREDICTED: transcription factor bHLH110 isoform X1 [*Malus domestica*]	3.17243 × 10^−5^	−1.456370629	down

**Table 2:** Differentially expressed b-ZIP transcription factors in LF and GLF fruit.

Gene ID	Gene Name	FDR (False Discovery Rate)	Log2FC	Relative Expression Mode
MD06G1217200	PREDICTED: transcription factor WER [*Malus domestica*]	9.53635 × 10^−18^	1.005209081	up
MD16G1228600	PREDICTED: transcription factor MYB21-like [*Pyrus × bretschneideri*]	2.60867 × 10^−5^	1.248571648	up
MD08G1031200	PREDICTED: protein RADIALIS-like 3 [*Malus domestica*]	1.42606 × 10^−5^	2.575760236	up
MD02G1308300	PREDICTED: peptidyl-prolyl cis-trans isomerase FKBP42-like isoform X2 [*Malus domestica*]	1.63394 × 10^−7^	2.073944629	up
MD11G1104500	PREDICTED: uncharacterized protein LOC108172356 [*Malus domestica*]	7.86998 × 10^−9^	3.492959082	up
MD14G1172900	PREDICTED: transcription factor MYB86-like [*Pyrus* × *bretschneideri*]	0.006193873	−1.831702439	down
MD16G1148300	PREDICTED: transcription factor MYB108-like [*Malus domestica*]	8.63823 × 10^−7^	−3.028824041	down
MD04G1184900	MYB domain class transcription factor [*Malus domestica*]	0.006696246	−1.001992516	down
MD02G1087900	PREDICTED: transcription factor TT2 [*Pyrus* × *bretschneideri*]	0.009579611	−1.076780594	down
MD14G1222200	PREDICTED: transcription factor DIVARICATA-like [*Pyrus* × *bretschneideri*]	0.000154883	−1.155980792	down
MD15G1025600	PREDICTED: protein RADIALIS-like 6 [*Malus domestica*]	5.1832 × 10^−5^	−1.671371068	down
MD16G1083300	PREDICTED: transcription factor MYB1R1 [*Malus domestica*]	3.09699 × 10^−5^	−1.155418453	down
MD05G1341500	PREDICTED: MYB-like transcription factor ETC1 [*Malus domestica*]	4.23716 × 10^−5^	−1.340022381	down
MD10G1216700	PREDICTED: myb family transcription factor APL-like [*Pyrus* × *bretschneideri*]	0.000537588	−1.042637231	down
MD11G1173100	PREDICTED: transcription factor LHW-like [*Pyrus* × *bretschneideri*]	2.90818 × 10^−5^	−1.014574381	down

**Table 3:** Differentially expressed bHLH transcription factors in LF and GLF fruit.

Gene ID	Gene Name	FDR (False Discovery Rate)	Log2FC	Relative Expression Mode
MD08G1123300	PREDICTED: basic leucine zipper 9-like [*Malus domestica*]	6.47336 × 10^−12^	1.431449022	up
MD03G1051900	PREDICTED: transcription factor RF2b-like [*Malus domestica*]	1.18702 × 10^−9^	1.264167539	up
MD02G1189300	PREDICTED: basic leucine zipper 8-like [*Malus domestica*]	2.35816 × 10^−5^	1.127923266	up
MD08G1123300	PREDICTED: basic leucine zipper 9-like [*Malus domestica*]	6.47336 × 10^−12^	1.431449022	up
MD03G1051900	PREDICTED: transcription factor RF2b-like [*Malus domestica*]	1.18702 × 10^−9^	1.264167539	up
MD02G1189300	PREDICTED: basic leucine zipper 8-like [*Malus domestica*]	2.35816 × 10^−5^	1.127923266	up

**Table 4:** Primers used in this study. The *Nde* I and *Sac* I sites used for vector construction are underlined.

Assay	Primer Name	Sequence (5′–3′)
Gene expression	MdAux/IAA2-ex-F	GAACTTGTTGTGGTGTGGAG
	MdAux/IAA2-ex-R	CGCTGATTGCTACTAAGCTG
	MdActin-F	GGCTGGATTTGCTGGTGATG
	MdActin-R	TGCTCACTATGCCGTGCTCA
Gene cloning	MdAux/IAA2-full-F	ATGGATAATTTGTATAGTATGAT
	MdAux/IAA2-full-R	TCATCTTCCAATTCCAACTT
	MdAux/IAA2-pro-F	AGATAGCTAAGAATTTCTGGCGTA
	MdAux/IAA2-pro-R	GCCCTTACAACTCCATTATTCTCA
Vector construction	pRI101-MdAux/IAA2-OE-F	TCTTCACTGTTGATACATATGATGGATAATTTGTATAGTATGAT
	pRI101-MdAux/IAA2-OE-R	CGATCGGGGAAATTCGAGCTCTCATCTTCCAATTCCAACTT
	pRI101-MdAux/IAA2-AN-F	CGATCGGGGAAATTCGAGCTCATGGACGGGAAGAAACTGGA
	pRI101-MdAux/IAA2-AN-R	TCTTCACTGTTGATACATATGCTAAGGGATCCACTTAGGATCT

## Data Availability

The data that support the findings of this study are available from the corresponding author upon reasonable request.

## Supplementary Materials

The following supporting information can be downloaded at: https://www.mdpi.com/article/10.3390/ijms23169454/s1.
