# Supplementary figures and images for: The MdAux/IAA2 Transcription Repressor Regulates Cell and Fruit Size in Apple Fruit
Source: Int J Mol Sci. 2022 Aug 21;23(16):9454. doi: 10.3390/ijms23169454 (PMC9408813; doi:10.3390/ijms23169454)

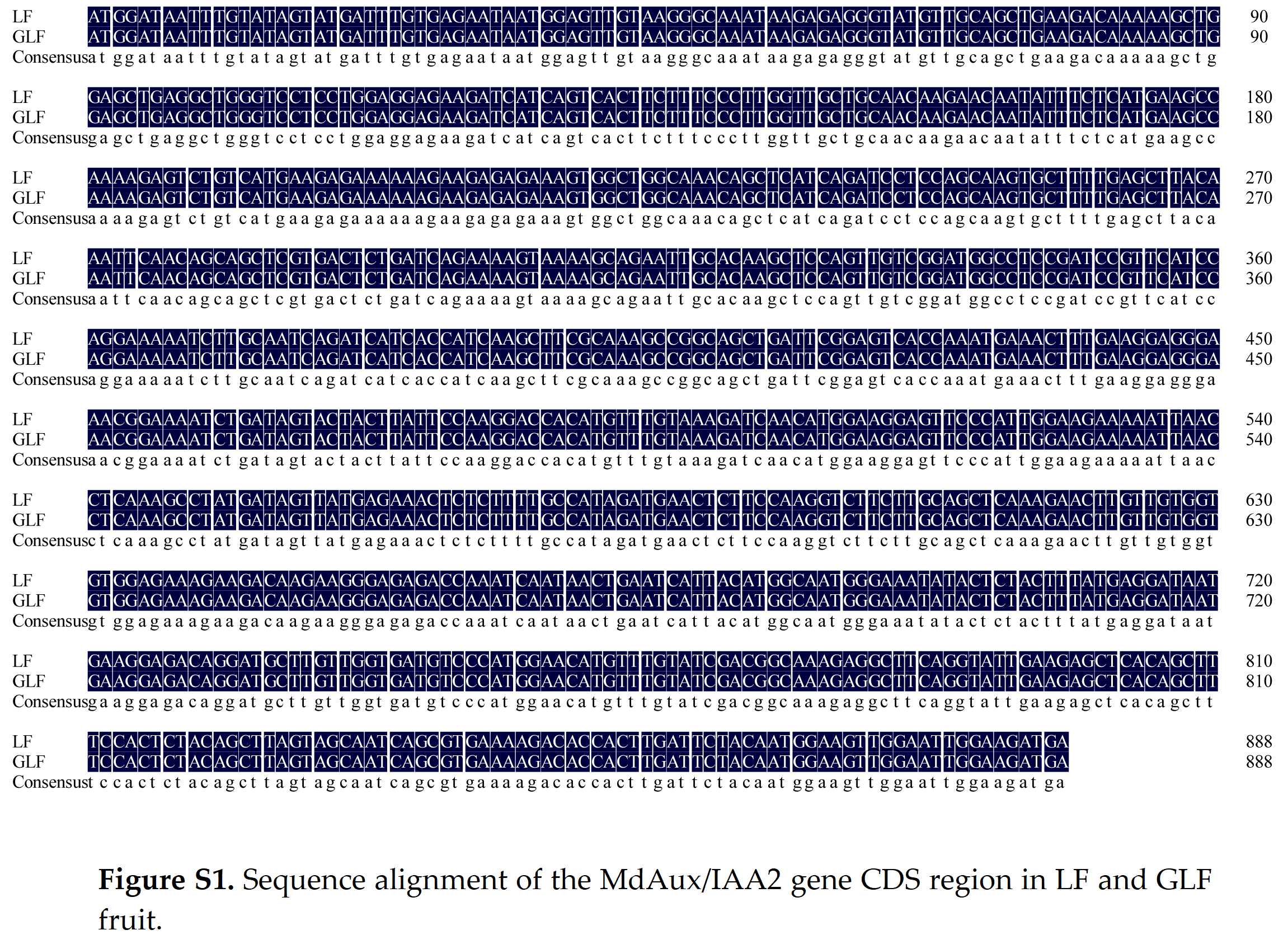

Supplement: Supplementary file 1 [file ijms-23-09454-s001.zip › Figure S1. Sequence alignment of the MdAuxIAA2 gene CDS region in LF and GLF fruit.tif]

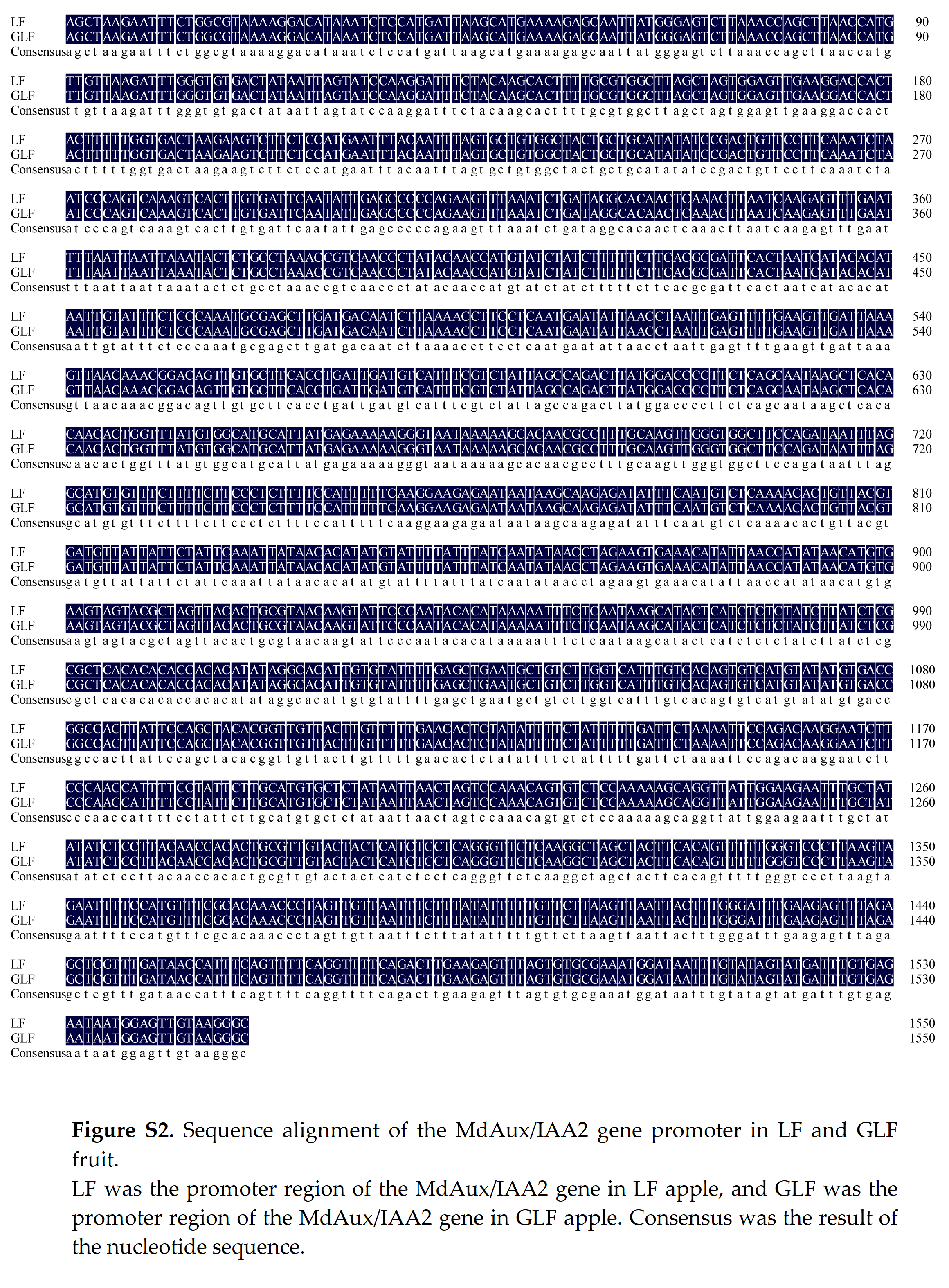

Supplement: Supplementary file 1 [file ijms-23-09454-s001.zip › Figure S2. Sequence alignment of the MdAuxIAA2 gene promoter in LF and GLF fruit.tif]

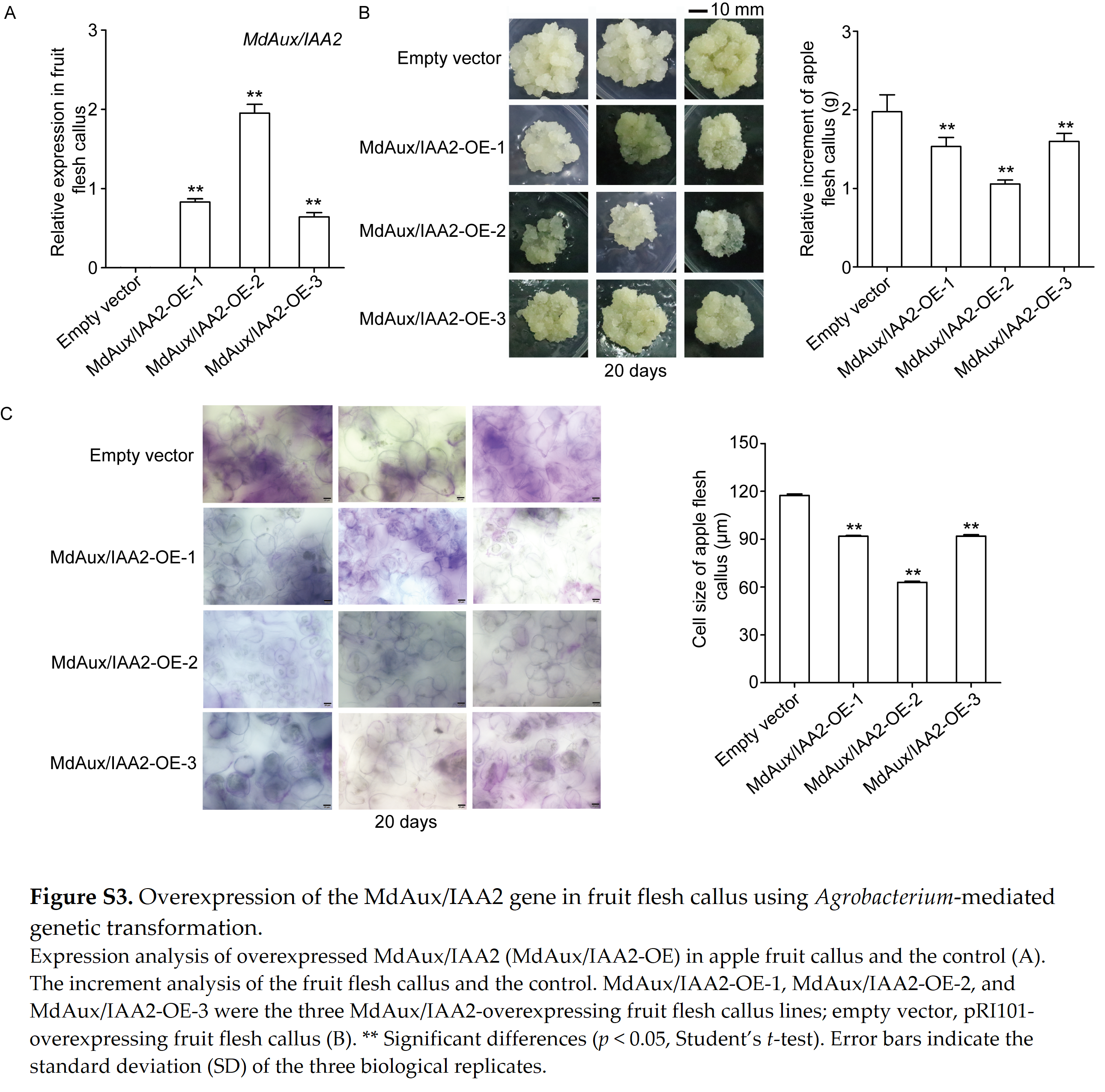

Supplement: Supplementary file 1 [file ijms-23-09454-s001.zip › Figure S3. Overexpression of the MdAuxIAA2 gene in fruit flesh callus using Agrobacterium-mediated genetic transformation..tif]

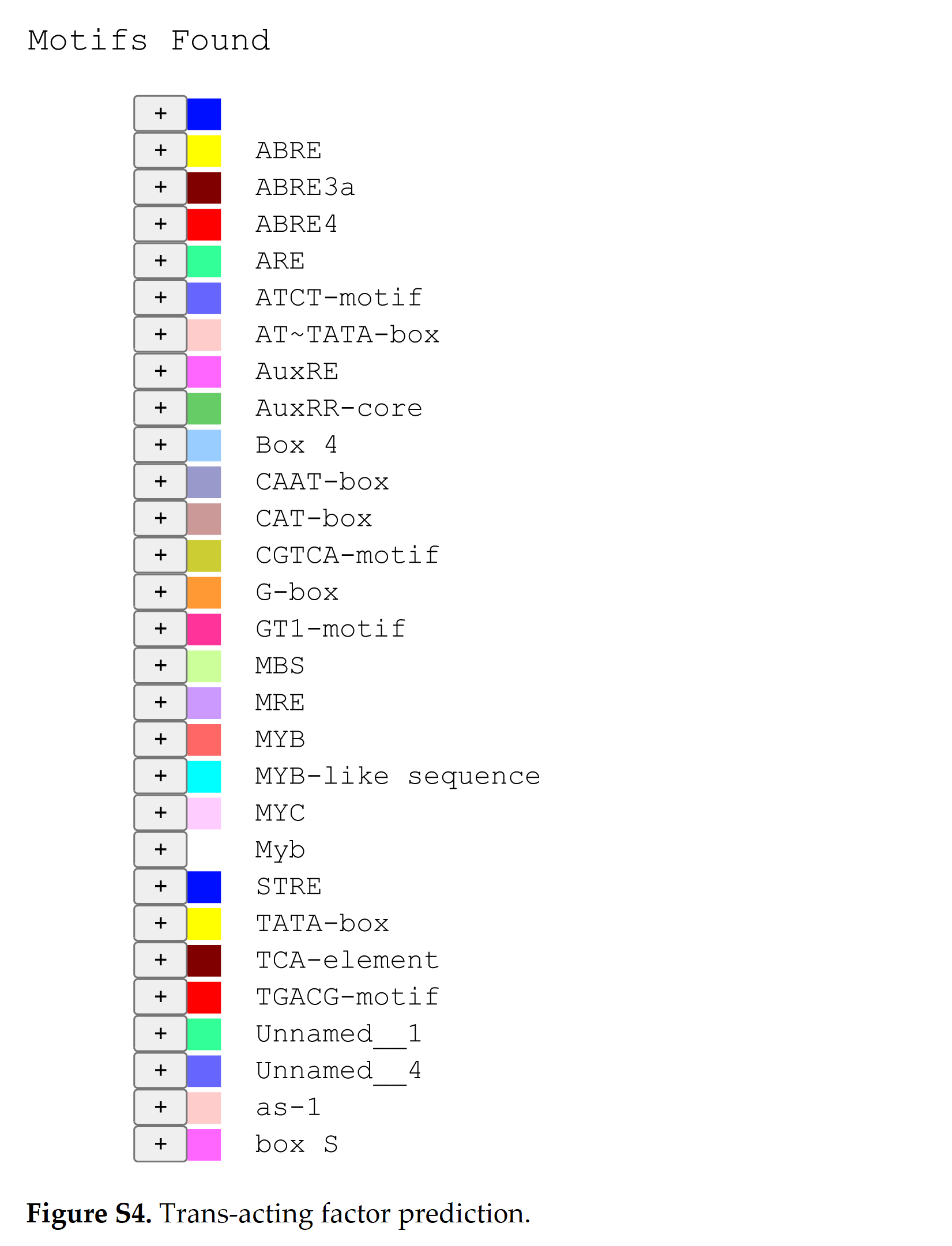

Supplement: Supplementary file 1 [file ijms-23-09454-s001.zip › Figure S4. Trans-acting factor prediction.tif]
